# Supplementary material for: Are carbon emissions trading and green financial instruments synergistic? -Comprehensive quantitative research based on content analysis
Source: PLoS One. 2024 Mar 7;19(3):e0298601. doi: 10.1371/journal.pone.0298601 (PMC10919601; doi:10.1371/journal.pone.0298601)
Supplement: S1 Data — (ZIP) [file pone.0298601.s001.zip › Data/地级市Z_F工作报告.zip!/║╙─╧/┬σ╤⌠╩╨/└┤╫╘╣1⁄2╓┌║┼í╛┬φ┐╦╩2╛▌═°í┐_2009.docx]

各位代表：

现在，我代表市人民政府向大会作工作报告，请予审议，并请各位政协委员和列席人士提出意见。

过去的五年，洛阳实现了跨越式发展

2004年以来的五年，是我市改革发展进程中极不平凡的五年。在省委、省政府和市委的正确领导下，在市人大、市政协的监督支持下，全市上下深入贯彻落实科学发展观，抢抓发展机遇，积极应对挑战，奋力开拓进取，经济社会不仅实现了跨越式发展，而且特色鲜明、亮点纷呈。

五年拼搏，综合经济实力快速提升。2008年，全市实现地区生产总值1919.6亿元，是2003年的2.8倍，年均增长15.5%。财政一般预算收入116.6亿元，是2003年的3.9倍，年均增长31%。全社会固定资产投资1105.5亿元，是2003年的5.2倍，年均增长39.1%。社会消费品零售总额577.1亿元，是2003年的2.5倍，年均增长17.6%。金融机构存款余额达到1395.2亿元，年均增长15.2%。综合经济实力居全国大中城市第43位、中部城市第4位。

五年实干，项目建设取得重大进展。一大批工业项目建成投产。五年投入产业引导资金2.3亿元，实施千万元以上工业项目3296个、完成投资1580.8亿元。洛阳石化油品质量升级改造、华润首阳山2×60万千瓦机组、香江万基120万吨氧化铝一二期、中铝河南铝业20万吨铝板带、中硅公司3000吨多晶硅、尚德公司120兆瓦太阳能电池、中集凌宇5000台客车及专用车等一批重大项目竣工投产。中信重工新重机工程、LYC新洛轴工程、一拖工业园、中铝洛铜10万吨高精度电子铜板带、725所5000吨钛板带、中硅公司新2000吨多晶硅、洛钼集团4万吨钼冶炼、阿特斯3000吨长晶及切片等一批重大项目顺利推进。在项目建设的带动下，我市传统产业不断升级，优势产业不断壮大，新兴产业初具规模。一大批服务业项目加快实施。商贸流通业实施千万元以上项目61个、总投资65亿元。房地产业完成投资578.7亿元、年均增长54.7%。丹尼斯、王府井等项目相继建成开业，宝龙城市广场等项目顺利推进。一大批交通基础设施项目如期竣工。五年累计投资112.7亿元，建成高速公路 124 公里 、干线公路 760 公里 、县乡公路 919 公里 、“村村通”道路 6944 公里 ，环城高速和南部山区大环线已经贯通；交通扶贫五年完成投资6.2亿元，建成农村公路 2544 公里 。一大批社会事业项目相继建成。河南科技大学、洛阳理工学院新校区等项目建成投用。7个贫困县、缺水县县城供水项目全部建成。投资4亿多元，提前两年在全省率先完成了农村中小学D级危房改造。乡镇卫生院改造顺利完工。

五年奋斗，结构调整取得明显成效。工业实现了超常规发展。2008年，工业经济占经济总量的比重达到54.5%，比2003年提高4.4个百分点。规模以上工业增加值783.3亿元，是2003年的4.1倍；主营业务收入2699.3亿元，是2003年的4.9倍；工业总量居全国中心城市第22位。装备制造、能源电力、石油化工、有色金属、材料工业等五大优势产业占规模以上工业增加值的比重达到75.1%。全市工业主营业务收入超亿元企业353家，其中超50亿元企业9家、超100亿元企业5家、超200亿元企业1家。新电集团、伊电集团、一拖集团跻身中国企业500强。我市被命名为新材料国家高技术产业基地，荣获2008中国制造业名城称号。旅游业实现转型升级。发挥历史文化和自然资源优势，加快景区建设和旅游产品开发，龙门石窟入选 5A 级旅游景区、全国文明风景旅游区，牡丹花会、河洛文化旅游节等节会的影响进一步扩大，“千年帝都、牡丹花城、丝路起点、山水洛阳”的城市品牌享誉海内外。2008年，共接待游客4567万人次、其中入境游客30.8万人次，分别是2003年的3.2倍和11.5倍；旅游总收入224亿元，旅游创汇9399万美元，分别是2003年的3.4倍和8.5倍。洛阳正大步向国际化旅游目的地城市迈进。创新能力不断增强。五年承担国家、省各类科技项目460项，其中国家“ 863” 计划和科技支撑计划26项。获国家、省科技进步奖119项，其中超薄浮法玻璃关键技术及装备研发、铝合金热连轧技术工艺开发获国家科技进步一等奖。市级以上各类研发机构已达170家。我市荣膺全国科技进步先进市、国家知识产权示范市称号。高新区成为全省第一家国家知识产权试点园区。节能减排成效显著。建立节能减排统计、监测和考核体系，实施碧水蓝天青山绿地工程，推动重点企业、重点行业、重点区域的节能降耗和环境整治。加快环保基础设施建设，9县（市）污水处理厂、垃圾处理场全部建成投用。市区空气质量二级以上天数达到336天。省定节能减排目标全面完成。深入开展资源整合和土地“三项整治”，资源集约节约利用水平不断提高。

五年建设，城市面貌焕然一新。第三期城市总体规划顺利实施，科学编制第四期城市总体规划和土地利用总体规划。洛阳新区迅速崛起。按照以洛河为轴线、南北对应发展的思路，高起点规划、大手笔建设洛阳新区。五年累计开工各类工程1580项，完成投资440亿元，新建道路96公里、景观水系60公里、绿地476万平方米，开发面积30平方公里，聚集人口25万。新区行政中心、体育中心已经形成，洛阳歌剧院、开阳湖等一大批标志性工程建成投用，新区博物馆、新区医院、新区高中等项目进展顺利。滨河百栋高层累计开工135栋。经过全市上下的不懈努力，一座靓丽的现代化新城区已矗立在洛河之滨。旧城改造步伐加快。五年累计竣工各类项目127个，完成投资200亿元，新建、改扩建了一大批城市道路、背街小巷、广场绿地，实施了一大批城中村、城郊村改造和河渠整治项目。城市基础设施建设不断加强。定鼎门污水处理厂等一批项目建成投用，机场改扩建、郑西铁路客运南站、凌波桥、瀛洲桥等一批项目顺利实施。生态建设成效显著。累计投入8亿元，实施退耕还林、通道绿化、环市区苗木花卉林果产业带、北邙生态屏障等工程，周山、上清宫、隋唐城遗址植物园等森林公园相继建成，全市森林覆盖率42.8%，城市绿化覆盖率38.3%。城市形象大幅提升。深入开展城市创建活动，搞好绿化、亮化、美化，加强精细化管理，我市先后荣膺国家卫生城市、中国十大最佳魅力城市、倾国倾城·最值得向世界介绍的中国名城、欧洲人最喜爱的中国十大旅游城市等称号。

五年努力，新农村建设全面推进。县域经济实力显著增强。2008年，县（市、区）生产总值占全市的比重达到75.9%，比2003年提高12.3个百分点。偃师市、栾川县、新安县、伊川县与汝阳县、洛宁县、宜阳县、嵩县先后入选全省县域经济综合实力强县和先进县，孟津县在全省县域经济排序中由2003年的46位上升到39位。县城改造和小城镇建设加快推进。偃师市、嵩县、栾川县等城乡一体化试点进展顺利。现代农业稳步发展。粮食总产连续六年超200万吨。畜牧业、林果业、烟叶、牡丹花卉等特色产业不断壮大。农业产业化水平不断提高。实施“农村百万劳动力转移”工程，年劳务输出由2003年的40多万人次发展到140多万人次。农业基础设施建设加快推进。五年完成水利投资近15亿元，西霞院水库、小浪底南岸引水、陆浑西干渠、龙脖灌区、新安提黄、禹门河反调节水库等一批重点工程建成投用，前坪水库前期工作全面展开。农村公共服务不断完善。分类推进新农村建设，积极实施“村村整治千村达标百村示范”工程，农村社区服务中心、通组通户道路等公共设施建设加快推进。解决农村安全饮水64万人。农村户用沼气由6.1万座增加到45万座。“万村千乡市场工程”和“家电下乡”工作加快推进。全市基本实现了行政村通电话、通自来水、通水泥路、通客车、通广播电视。

五年探索，各项改革步伐加快。国企改革实现重大突破。加快国企战略重组，先后引进中铝公司、中钢集团、中集集团、中建材、中色总院、永煤集团等40多家优强企业，引进资金210亿元，盘活资产144亿元，国有经济控制力、影响力和带动力明显增强。先后有4家企业4支股票上市，募集资金89.6亿元。市属203户中小企业改革基本完成。其他领域改革不断加快。乡镇综合机构改革全面完成。行政管理体制和事业单位改革不断深化。财政部门预算、收支两条线、国库集中支付、政府采购等制度进一步完善。公路、公用、市政等领域改革迈出重要步伐。

五年开拓，对外开放不断深入。招商引资成效显著。五年共引进千万元以上项目1387个，总投资2448亿元。审批外商投资企业220家，实际利用外资19.5亿美元、市外境内资金588亿元，分别是上个五年的2.2倍、8.1倍和5.6倍。家乐福、沃尔玛、台塑集团等5家世界500强企业，中船重工、恒大地产等30家国内500强企业入驻我市。对外贸易成果丰硕。2008年，全市完成进出口总额18.9亿美元；其中出口13.6亿美元，是2003年的4.1倍。非公有制经济发展突飞猛进。2008年，非公有制经济完成营业收入、增加值、上缴税金分别是2003年的5.1倍、6.1倍和10.2倍，对全市经济的贡献率由2003年的28.7%提高到50.6%。

五年投入，民生质量得到新提高。人民生活不断改善。在办好省定实事的同时，坚持每年为城乡居民办好二十件实事。五年市级财政用于民生领域的支出达260.4亿元，占财政一般预算收入的65%以上。全市贫困人口由2003年的42.9万人下降到23.4万人。2008年，城镇居民人均可支配收入和农民人均纯收入分别达到14672元和4597元，比2003年增长1.9倍和2倍。社会保障体系日臻完善。五年新增城镇就业66.7万人，发放小额担保贷款3.8亿元。企业退休人员养老金、最低工资标准、城乡低保和五保户供养标准不断提高。城镇职工基本医疗保险、城镇居民基本医疗保险、新型农村合作医疗、城乡困难群众医疗救助等四大制度互为补充、覆盖城乡的医疗保障体系初步建立。以廉租住房、经济适用房、住房公积金为重点的住房保障体系不断完善。

五年积聚，社会事业全面进步。教育、卫生、文化等事业加快发展。认真落实“两免一补”资助政策，五年市财政用于教育的投入达到94.3亿元，是上个五年的2倍。小学、初中适龄儿童入学率分别达到100%和99.7%。我市代表河南省顺利通过国家“两基”验收。普通高招连创佳绩。职业教育稳步发展。成功组建洛阳理工学院。乡镇卫生院、村卫生室、社区卫生服务站等基层卫生服务网络不断完善，突发公共卫生事件防控能力明显提高。城乡文化活动蓬勃开展，涌现出了《河洛风》等一批优秀作品。河洛大鼓等7个项目入选国家非物质文化遗产名录。洛阳片区大遗址保护工程被列为国家大遗址保护重大项目，隋唐城遗址、汉魏故城整体保护规划已经完成，偃师商城、汉魏故城等保护展示工程初见成效，隋唐城宫城核心区等保护展示工程加快推进。丝绸之路和大运河洛阳段申报世界文化遗产工作取得阶段性进展。地方史志工作继续走在全省前列。各大公园免费开放。全民健身运动广泛开展。人口自然增长率稳定控制在6‰以内。民族宗教、外事侨务、新闻出版、统计、审计、人防、地震、气象、残疾人事业等工作都取得了新成绩。精神文明和民主法制建设不断加强。广泛开展“讲正气、树新风、建绩效”教育活动和“新解放、新跨越、新崛起”解放思想大讨论活动，全市人民精神面貌焕然一新。驻洛部队和武警部队为地方经济社会发展做出了重要贡献。我市荣获全国双拥模范城五连冠。自觉接受人大及其常委会的法律监督、工作监督和政协的民主监督，人大代表议案、建议和政协委员提案办结率100%、满意率98%以上。五年提请市人大及其常委会审议地方性法规22件，发布行政规章60件。“五五”普法教育深入开展。严查违法乱纪案件，党风廉政建设和反腐败斗争取得新成效。始终把稳定放在重中之重的位置，安全生产、社会治安和信访工作成效显著，社会大局基本稳定。

刚刚过去的2008年，洛阳更是在挑战中奋力前行。面对雨雪冰冻灾害、汶川特大地震、国际金融危机带来的困难和压力，全市上下迎难而上，积极应对，经济社会继续保持平稳较快发展，在洛阳改革发展史上谱写了令人难忘的一页。加强协调服务，经济运行总体平稳。面对急剧变化的经济形势，及时调整工作重点，加强经济即期调节，搞好生产要素衔接，积极为企业排忧解难。全年为企业退税、减税42亿元；组织银企洽谈会4次，签约资金267.6亿元。抢抓发展机遇，着力夯实发展基础。紧紧抓住中央新增1000亿元投资等机遇，围绕国家重点支持的领域，全市上下积极行动，筛选、申报重大项目78个，争取中央投资4.3亿元。狠抓项目建设，支撑能力不断增强。全年实施千万元以上项目2131个、完成投资773亿元。其中工业项目1354个、完成投资539亿元，中信重工冶铸基地等1106个项目竣工投产；伊电集团50万吨连铸连轧等112个项目加快推进；轴研科技精密重型机械轴承等1130个项目开工建设；华阳孟津电厂、洛阳石化45万吨PX等839个项目获国家、省、市核准备案；新奥薄膜太阳能电池、南车集团轨道交通装备等项目前期积极推进；超前谋划了河柴重工新能源气体发动机、尚德公司1000兆瓦太阳能电池组件等260个重大项目，总投资超过5000亿元。高度重视“三农”工作，农村经济健康发展。积极落实粮食直补、农机具购置补贴等一系列惠农政策，全年粮食总产230.8万吨，创历史新高。孟西灌区工程即将竣工，9座水库除险加固工程顺利实施。建成搬迁安置点55个、安置贫困人口万余人，搬迁扶贫工作经验在全国推广。深化改革开放，外向型经济实力进一步壮大。国机集团与一拖集团成功重组，第一批用于一拖新项目建设的17亿元资金已于 2 月 28 日 到位。我市全年实际利用外资9亿美元、市外境内资金230.6亿元，分别增长41.3%和35.5%。中信银行、兴业银行、光大银行、长城保险等入驻洛阳。我市成为国家中西部加工贸易重点承接地。着力改善民生，解决了一大批关系群众切身利益的问题。市级财政投入5.8亿元，省定、市定实事圆满完成。就业再就业成效显著，社会保障体系和住房保障体系不断完善，各项社会事业取得新成绩。同时，全力做好抗震救灾和对口援建工作，成功组织了奥运会和残奥会火炬洛阳传递活动。

各位代表！过去的五年，是改革开放以来我市综合实力增长最快的五年，是城乡面貌变化最大的五年，是人民群众得到实惠最多的五年，是城市知名度和美誉度提升最快的五年，是社会稳定和谐的五年，一个充满活力、更具魅力的美好洛阳正呈现在世人面前。五年来，我们始终坚持解放思想、与时俱进。敢于冲破旧的观念束缚，大胆探索、勇于实践，创造性地提出了实施“工业强市、旅游强市、科教强市”三大战略，再造一个新洛阳、全面走在中原崛起前列的发展目标。始终坚持抢抓机遇、科学发展。牢牢把握发展这个第一要务，一刻也不放松地抓住项目建设这个载体，突出抓好结构调整、科技创新和节能减排，着力转变发展方式，发展能力不断提高。始终坚持深化改革、扩大开放。深化重点领域和关键环节改革，推进体制机制创新，加大招商引资力度，发展后劲明显增强。始终坚持以人为本、促进和谐。始终把实现好、维护好、发展好最广大人民的根本利益，作为一切工作的出发点和落脚点，认真解决好民生问题，加强和谐洛阳建设，让更多发展成果惠及广大人民群众。始终坚持依法行政、勤政为民。依法履行政府职能，规范行政行为，全面提高政府执行力，为人民群众服务的能力不断提高。

回顾这浓墨重彩的五年，我们为洛阳发生的巨大变化而骄傲，为全市人民创造的辉煌业绩而自豪！这是省委、省政府和市委正确领导的结果，是市人大、市政协和社会各界监督支持的结果，是全市人民团结奋斗的结果。在此，我代表市人民政府，向辛勤工作在各条战线的全市人民，向人大代表、政协委员，向各民主党派、人民团体，向离退休老领导、老同志，向驻洛部队、武警官兵和公安干警，向所有关心支持洛阳发展的社会各界和海内外朋友，表示衷心的感谢并致以崇高的敬意！

在总结成绩的同时，我们也清醒地看到，我市经济社会发展中还存在一些薄弱环节和突出问题：科学发展的理念需要进一步明晰。思想解放还不到位，开拓创新意识还需要增强，科学发展的能力有待进一步提高。结构调整的步伐需要进一步加快。产业结构不尽合理，第三产业比重逐年下降，且增速连续5年低于GDP增速。农业基础依然薄弱，现代农业总量偏小，农民持续增收难度加大。工业增长方式粗放，资源性、初加工型产业比重大，节能减排任务重；产业链条短，产业集中度不高，研发投入比重低于全国、全省平均水平，企业核心竞争力不强。文化资源优势尚未充分发挥，现代服务业发展缓慢。制约发展的体制机制性障碍需要进一步破除。改革攻坚的任务仍十分繁重，对外开放的深度和广度需进一步拓展。民生方面存在的薄弱环节需要进一步加强。就业形势依然严峻，社会保障压力较大，教育、医疗、住房等群众关心的热点难点问题还没有很好地解决，困难群体的救助帮扶机制还需要加快完善。政府职能和机关作风需要进一步转变。政府职能转变还不到位，个别部门和机关工作人员大局意识、服务意识不强，经济发展环境需进一步优化。对此，我们一定高度重视，采取有力措施，认真加以解决。

未来的五年，我们面临着新形势新任务

今后五年是我市全面完成“十一五”规划、科学编制和加快实施“十二五”规划、为全面实现小康社会宏伟目标奠定坚实基础的五年。当前，国际金融危机对实体经济的影响正在加深，全球经济增长明显减速，国内经济下行压力不断加大，发展的不确定因素明显增多。同时，我们也要看到，我们仍处在一个重要的战略机遇期，经济发展的基本态势并未因金融危机的影响而逆转。全球经济调整必将促进国际国内产业加速转移，有利于我们扩大开放，借力发展；危机形成的倒逼机制压力，有利于我们加快结构调整和产业升级；国家实行积极的财政政策和适度宽松的货币政策，有利于我们争取更多的支持；特别是经过 30年的改革开放，我市综合实力显著提升，市场经济体制不断完善，为今后的发展奠定了坚实的思想基础和物质基础。挑战前所未有，机遇也前所未有。我们必须科学分析面临的新形势，积极顺应人民群众的新期待，凝心聚力，加快发展，努力实现全面走在中原崛起前列的宏伟目标。

今后五年经济社会发展的主要目标是：

——综合实力显著增强。在优化结构、提高效益、降低消耗、保护环境的基础上，到2013年，力争全市生产总值、财政一般预算收入、全社会固定资产投资比2008年翻一番。

——经济结构明显优化。转变发展方式取得实质性进展；特色农业加快发展，农业产业化占农业总产值的比重达到45%以上；工业优势更加突出，五大优势产业占规模以上工业增加值的比重达到80%以上，高技术产业占规模以上工业增加值的比重超过5%；服务业水平不断提升，第三产业占地区生产总值的比重达到32%。自主创新能力不断增强，创新型城市建设取得明显成效。

——城乡发展更趋协调。城乡居民生活水平和质量不断提高，绝对贫困现象基本消除。城镇居民人均可支配收入年均增长8%以上，农民人均纯收入年均增长6.5%以上。城市功能更加完善，文化特色更加突出，集聚能力和带动作用更加明显。全市城镇化率达到50%以上，城乡一体化发展格局逐步形成。

——发展活力不断增强。国企改革进一步深化，现代企业制度更加完善；其他重点领域改革取得新成效。经济外向度明显提高，到2013年，实际利用外资15亿美元以上，引进市外境内资金1000亿元以上；外贸出口24亿美元。非公有制经济比重达到58%。市场体系更加完善，发展环境进一步优化。

——生态环境更加宜人。资源节约型、环境友好型社会建设取得明显成效。万元生产总值能耗下降16%以上，化学需氧量、二氧化硫排放量分别下降10%和30%。城市污水处理率、垃圾处理率分别达到90%和98%。城市空气质量优良天数稳定在300天以上。森林覆盖率和城市绿化覆盖率均达到45%，生态环境明显改善。

——社会建设不断进步。覆盖城乡的社会保障体系日趋完善。劳动就业更加充分，新增城镇就业60万人。教育、卫生、文化等社会事业全面发展，民主法制不断加强，文明程度明显提高，和谐洛阳建设全面推进。

实现上述目标，必须科学分析我市的比较优势，理清工作思路，突出工作重点，把握关键环节，一以贯之地加以推进：一是坚定不移地加快工业发展，靠工业增强实力。继续实施工业强市和项目引资双带动战略，积极谋划建设一批打基础、管长远、增实力的工业项目，提高企业创新能力，推动产业优化升级，不断增强综合竞争力。二是坚定不移地加快城市建设，靠城市提升形象。高标准建设“洛阳新区”，加快洛北城区改造，进一步拉大城市框架，完善城市功能，改善城市环境，不断提升城市的承载力和美誉度，努力把洛阳建成山水园林相间、生态环境优美、人民生活舒适的最佳人居环境城市。三是坚定不移地加快文化旅游业发展，靠文化彰显特色。充分发挥洛阳丰富的历史人文资源优势，发展壮大文化旅游业。在城市建设中更多地融入历史文化元素，进一步延续历史文脉，彰显城市个性，着力打造国际知名城市品牌。四是坚定不移地抓好“三农”工作，靠农业稳固基础。认真贯彻落实党的十七届三中全会精神，坚持把解决好“三农”问题作为重中之重，加大农业基础设施建设力度，加快农业结构调整，千方百计增加农民收入，努力实现农业和农村经济持续健康发展。五是坚定不移地转变发展方式，靠调整促进转型。按照农业优质高效、工业优化升级、服务业优强发展的思路，努力在优化结构、创新发展等方面实现新突破，促进经济增长由主要依靠第二产业带动向三次产业协调发展转变。六是坚定不移地推进科技创新，靠科技提升竞争力。发挥洛阳的科研优势，加大研发投入，加强产学研结合，加快科技成果就地转化，大力发展高新技术产业，营造良好的创新环境，不断提高科技进步对经济增长的贡献率。七是坚定不移地把改善民生作为一切工作的出发点和落脚点，靠改善民生促进和谐发展。集中财力、物力，建成一批群众真正得到实惠的民心工程，解决好关系人民群众切身利益的热点难点问题，激发全市人民的积极性和创造性，促进社会和谐发展。

2009年，我们要全力保持经济社会平稳较快发展

2009年是新一届政府的开局之年。政府工作的总体要求是：全面贯彻党的十七大、中央经济工作会议和省委八届九次全会、省十一届人大二次会议精神，深入学习实践科学发展观，积极顺应国家宏观调控政策，以保持经济平稳较快发展为政府工作的首要任务，抢抓机遇，应对挑战，共克时艰，大力实施项目引资双带动战略，加快转变发展方式，继续深化改革开放，认真解决关系群众切身利益的热点难点问题，促进经济社会又好又快发展。

经济社会发展的主要预期目标是：地区生产总值增长11%左右；地方财政一般预算收入增长9%左右；全社会固定资产投资增长20%左右；社会消费品零售总额增长15%左右；外贸出口不断增长；城镇居民人均可支配收入增长7%，农民人均纯收入增长6%；城镇新增就业人数10万人，登记失业率控制在4.5%以内；万元生产总值能耗下降4.84%；居民消费价格涨幅控制在5%以内；人口自然增长率控制在6.5‰以内。

今年有可能是新世纪以来我市经济发展最为困难的一年，经济下滑已经成为制约我市加快发展的主要矛盾。我们必须增强抓机遇、谋发展的意识，充分做好打硬仗、攻难关的准备，努力营造负重奋进、加快发展的良好氛围。一是处理好保持经济增长与转变发展方式的关系。把扩内需、保增长与调结构、促转型紧密结合起来，依托产业集聚区建设，着力构建现代产业体系、现代城镇体系、自主创新体系，努力实现速度与结构、质量、效益的统一。二是处理好应对当前危机与谋划长远发展的关系。坚持远近结合、统筹兼顾，既要立足当前应对危机，办急事、抓大事，又要着眼长远谋划发展，强基础、增后劲，把应对危机的过程变成培育新的经济增长点的过程、变成推动科学发展的过程。三是处理好发展改革与保持稳定的关系。既要通过加快发展、深化改革解决人民群众关心的热点难点问题，以发展保稳定，又要创造和谐稳定的社会环境，以稳定促改革、促发展。四是处理好自力更生与借力发展的关系。既要充分挖掘自身潜力，苦练内功、艰苦创业，不断增强自我发展能力，又要眼睛向外，引进更多的资金、项目、技术、人才，借用外力加快发展。五是处理好扩大消费与改善民生的关系。既要把扩大消费作为改善民生、增加就业和社会保障的重要措施，又要把保障和改善民生作为扩大消费、促进增长的出发点、落脚点，切实提高人民群众生活水平。重点抓好以下工作：

（一）坚持投资拉动，不断增强经济发展后劲。加强项目建设、保持投资拉动，是促进经济平稳较快发展的最有效、最直接的措施。要紧紧围绕国家产业振兴规划、围绕构建现代产业体系，加快推进项目建设。突出抓好列入国家投资计划的项目。认真落实重点项目协调推进机制，加快项目建设进度，确保早建成、早见效。积极谋划一批事关长远发展的重大项目。加快吉利60万吨PTA、永骏新能源100万吨二甲醚、南车集团轨道交通装备、河柴重工船用高速大功率柴油机等重大项目前期工作。紧紧围绕国家5000亿元投资计划，抓紧谋划、申报一批技术水平高、带动能力强的项目。加快建设一批重点项目。工业方面计划实施千万元以上结构调整项目280个，总投资903.6亿元，当年完成投资370亿元。加快LYC新洛轴工程、华阳孟津电厂2×60万千瓦机组等140个项目建设，新开工洛阳石化260万吨柴油加氢和14万吨聚丙烯、725所风电叶片、伊电集团2×60万千瓦机组等140个项目，确保中信重工锻造基地、洛钼集团4万吨钼冶炼、中硅公司新2000吨多晶硅等项目如期竣工。能源方面要开工西气东输二线洛阳支干线等项目；加快120万吨正村煤矿、新义煤矿等项目建设，确保年底前投产；做好核电项目前期准备工作。交通方面要完成投资80亿元，重点抓好连霍高速洛阳段扩宽改造，郑西铁路客运专线洛阳段，洛宜铁路改线，偃师至洛阳、洛阳至吉利快速通道等项目；加快洛阳机场改扩建，确保候机楼主体工程花会前完工。做好石人山至栾川高速、洛阳至栾川高速前期准备工作。抓紧谋划洛阳城市轻轨、郑洛城际轨道交通等项目。进一步扩大政府投资。将原计划后三年政府投资的基础设施、公共设施项目，集中到今年安排，加快廉租住房、公共卫生、教育文化等设施建设，形成项目建设高潮。全年计划实施项目900个，带动全社会固定资产投资1200亿元以上。

（二）积极扩大消费需求，不断增强消费对经济的拉动作用。认真落实国家一系列扩内需的政策措施，大力发展服务业，开拓消费市场。提高居民消费能力。推进事业单位工资制度改革，落实企业最低工资制度，健全农民工工资支付保障机制。落实优抚对象、建国前老党员等抚恤和生活补助政策、困难群众社会救助政策，再次提高企业退休人员基本养老金、城乡低保标准和农村五保集中供养标准。加快发展生活性服务业。加快八角楼金街、万达建业广场等项目建设，积极引进优强商业企业入驻我市。大力发展社区服务、文化娱乐等吸纳就业能力强的生活性服务业。加快发展新区服务业，重点抓好关林市场改造、丹尼斯等商贸设施建设；完善新区公交体系，增设各类服务网点，方便居民生活。加快推进“万村千乡市场工程”和“家电下乡”工作，支持商业连锁企业向农村延伸。做大做强旅游业。办好2009世界邮展暨第27届洛阳牡丹花会；加快伏牛山生态旅游开发、玄奘故里二期、西霞院景区等重点旅游项目建设；积极开发具有文化内涵、地域特色和产业基础的旅游商品，努力打造区域旅游中心城市和国际性旅游目的地城市。力争全年接待游客5024万人次，其中入境游36万人次，旅游总收入246亿元。稳步发展房地产业。认真落实国家、省促进房地产业健康发展的各项政策，对资信好的房地产企业给予政策扶持。力争完成房地产开发80亿元，商品房施工面积1200万平方米，竣工350万平方米。

（三）加强经济运行协调，深入开展“企业服务年”活动。千方百计支持企业，努力在保生存、保市场、保发展上下工夫，确保经济不出现大的回落。加强运行监测调度。认真研究金融危机给企业带来的影响，准确把握产业和市场走势，及时为企业提供预警信息。切实加强生产要素的供需衔接，确保企业特别是重点企业的正常生产。加强分类指导。鼓励生产经营形势好的企业抓住调整机遇，努力抢占更大的市场空间；生产经营暂时遇到困难的企业，千方百计多拿订单，力保市场份额不减少；资源类企业加快资源整合，组建企业集团或产业联盟，拉长产业链条；产能过剩、技术相对落后的企业，一方面要加快技术改造保生存，一方面要通过兼并联合、破产重组，寻求新的生机。努力为企业排忧解难。安排1.2亿元产业引导资金，支持企业结构调整、技术改造和项目建设。利用增值税转型和设备价格走低的时机，引导企业加快更新关键技术装备，提升产品附加值和市场竞争力。认真落实税收优惠、缓缴社保费、降低保险费率、增加社保补贴等政策，切实减轻企业负担。建立重点企业和重点项目沟通联系制度，做好重点项目建设环境专项治理工作，及时帮助企业解决问题。

（四）加快转变发展方式，提高经济发展质量。优化资源配置，加快产业升级，着力构建工业、服务业相互支撑、互动发展的现代产业体系。发展壮大工业优势产业。围绕五大优势产业，在硅材料及光伏、光电技术、生物制药、节能环保等领域实施一批高技术产业化专项，形成一批特色产业集群。完善洛阳动力谷发展规划，抓紧启动一批重大项目，尽快形成新的产业优势。加快发展现代服务业。完善扶持政策，扩大规模，优化结构，不断提高现代服务业占三产的比重。大力发展现代物流、设计策划、技术研发等生产性服务业，鼓励大中型企业、科研院所二、三产业分离，提高核心竞争力。加快洛龙科技园区软件园、中储物流园区等重点项目建设。大力发展产业集聚区。按照突出特色、培育集群、形成链条、实现循环的原则，抓紧编制17个省级产业集聚区总体规划和控制性详细规划，出台扶持产业集聚区建设的政策措施，引导重点项目优先向集聚区布局，加强基础设施建设，培育优势企业，增强产业集聚功能，打造区域经济增长极。提高自主创新能力。加大研发投入，新建研发中心20家，实施产学研合作项目200个。加大新产品开发力度，重点突破高效晶体硅太阳能电池产业化、兆瓦级风力发电轴承、铁路桥梁橡胶支座等60项关键技术。支持有条件的高等院校、科研机构和企业建立国家、省重点实验室和工程中心。加快知识产权示范工程建设。做好高新技术企业认定和国家、省研发中心的申报工作。大力实施质量兴市战略，培育更多的名牌产品。加快推进节能减排。严格实行节能减排问责制和一票否决制，确保完成省定节能减排目标。实施新电集团电机系统、万基铝业窑炉余热利用等项目，推进公共机构节能、政府节能和工程节能。严格项目环境准入，防止低水平重复建设和盲目扩张。加大环境执法力度，集中开展违法排污专项整治。加快企业排污在线监控、污水处理厂自动监控、污水管网等基础设施建设。力争城市环境空气质量优良天数310天以上。大力发展循环经济。搞好循环农业示范市和节水型试点城市建设，在重点领域、重点行业建设一批资源综合利用项目。加强矿产资源整合，提高资源利用效率。加强土地监管，优先保障重大项目建设用地，切实保护耕地和基本农田。建成标准化厂房40万平方米。加强生态环境建设。积极创建国家森林城市，力争通过国家验收。抓好生态林、生态廊道、环市区苗木花卉林果产业带等工程，完成造林57.7万亩、中幼林抚育和低效林改造21.1万亩。加快生态洛河建设步伐。

（五）切实加强“三农”工作，促进农村经济健康发展。高度重视“三农”工作，加大政策支持力度，加强农业基础设施建设和新农村建设，促进农业稳定发展、农民持续增收。加大对“三农”的投入。认真落实各项惠农政策，加强农民负担监督管理。全年市财政安排“三农”资金2亿元，比上年增加2183万元。大力发展特色农业和品牌农业。加快孟津、伊川国家优质粮食产业工程基地县建设，全年粮食总产稳定在200万吨左右。加快发展现代畜牧业，新增肉制品加工能力10万吨、乳制品加工能力3万吨。支持本地乳制品企业占领市场，打响洛阳乳业品牌。积极发展烟叶、中药材、花卉苗木、林果等特色产业。新发展蔬菜基地5000亩。大力培育农产品深加工企业，争取有20家企业达到省级龙头企业标准；新发展各类农民专业合作社40家。完成农民工培训20万人次，劳务输出135万人次，创收70亿元以上。探索建立地方性农业保险体系。加强农田水利基础设施建设。加快前坪水库前期工作，开工建设15座中小型水库除险加固工程。抓好灌区修复配套节水改造，新增有效灌溉面积3万亩、节水灌溉3万亩，改造中低产田5万亩，治理水土流失200平方公里。加快新农村建设。建好200个示范村和600个重点整治村。基本完成中心村规划设计；硬化通组通户道路 2000 公里 ；新建农村社区服务中心200个；新发展农家店350个；新建农村户用沼气5万座、各类沼气工程50座、全托服务试点500个；解决20万人安全饮水问题；抓好100个贫困村整村推进，完成2250户1万人的扶贫搬迁任务。

（六）全面推进改革开放，进一步增强发展活力。加快体制机制创新，拓展对外开放的深度和广度，力求在重点领域和关键环节取得突破。加快企业兼并重组和改革改制。鼓励关联产业、上下游企业联合重组，提高抗风险能力。积极培育拥有自主知识产权和知名品牌的企业集团，提高产业集中度和资源配置效率，增强市场竞争力。抓好一拖集团战略重组，完成增资扩股。稳步推进洛轴集团、河柴集团、春都集团、春都集团股份政策性破产重组和物产集团、明珠热电等企业破产改制工作。解决好市属中小企业改制遗留问题。加强国有资产监管，提高监管效能和运营效益。深入推进其他领域改革。积极稳妥地推进燃油税改革、医疗卫生体制改革和集体林权制度改革。加强土地承包经营权流转管理和服务，发展适度规模经营。大力开展招商引资。发挥企业、产业、科技、资源、区位、环境等优势，围绕高新技术产业、高成长性关联产业、现代服务业、文化旅游业、农产品加工业等，抓紧谋划一批新的招商项目。抓住国际、国内产业转移机遇，积极开展重点产业、重点企业、重点区域的招商活动，引导电子、家电、服装、玩具等产业向我市转移。组织好2009世界邮展暨第27届洛阳牡丹花会、河洛文化旅游节等经贸活动，积极参加中博会、豫商大会、中国国际投资贸易洽谈会等重大招商活动，力争全年实际利用外资、市外境内资金分别增长13%和8%。进一步扩大外经外贸。用足用好外贸发展资金，重点支持企业开拓国际市场。落实国家鼓励出口政策，积极争取配额，扩大出口规模。优化出口结构，扩大机电产品、高新技术产品和深加工产品的出口。加快制订服务外包发展规划，积极承接发达国家和地区的服务外包业务。支持有条件的企业组成竞争力强的联合体，参与国际工程承包。鼓励紧缺资源能源、先进技术和关键设备进口。发展壮大县域经济。偃师市、栾川县、新安县与汝阳县、洛宁县、宜阳县要努力保持全省县域经济综合实力强县和先进县地位，伊川县和嵩县要争取再次进入全省县域经济综合实力强县和先进县行列，孟津县要争取在全省县域经济排序中位次前移。大力发展非公有制经济。认真落实各项扶持政策，鼓励非公有制企业参与国有资本调整和战略重组，引导资金、技术、人才等要素向优势企业集聚，推动企业上规模、上水平。积极搭建服务平台，完善非公有制企业服务体系。力争非公有制企业新上千万元以上项目290个，其中超亿元项目50个；营业收入超亿元企业达到130家。

（七）着力构建现代城镇体系，加快城乡一体化进程。实施中心城市带动战略，加快第四期城市总体规划和土地利用总体规划修编，加强基础设施建设，促进城乡协调发展。加快洛阳新区建设。搞好规划、完善机构、强化责任，努力将洛阳新区建成全省经济发展的重要增长极、改革发展的综合试验区和国际旅游精品区。全年实施项目111个、完成投资80亿元。重点抓好瀛洲桥、凌波桥、郑西铁路客运南站、洛阳呼叫产业园、新区医院、新区高中、新区污水处理厂、公交停车场等基础设施建设，加快沿河百栋高层、开元大道两侧组团开发。加快旧城改造步伐。全年实施项目155个，完成投资56亿元。重点抓好九都东路大修、道北二路、华山北路、瀛洲北路、滨河路、西苑桥改扩建、东出口排水管网、氵廛河治理等基础设施建设，开工建设洛浦公园西延伸工程。扩大以奖代补范围，加快南村、东涧沟、西小屯、五女冢等城中村、城郊村改造步伐，全年完成投资38亿元，新开工234万平方米。加强城市精细化管理。创新城市管理体制，提高城市管理水平。深入开展爱国卫生运动，巩固创建国家卫生城市成果。加强城市交通综合整治，切实缓解交通拥堵现象。加快城乡一体化进程。加快县城改造和小城镇建设，拆迁城中村120万平方米、开工建设250万平方米。完善优惠政策，积极推进户籍、就业、社会保障等制度的配套改革，促进农民向城镇集中，向二、三产业集聚。重点抓好偃师市、嵩县、栾川县城乡一体化试点工作。

（八）加快文化强市建设，彰显洛阳城市特色。发挥文化资源优势，推动文化发展与繁荣，进一步提升城市软实力。加强历史文脉保护。注重历史文化名城保护、开发和利用，加快偃师商城、汉魏故城和隋唐城宫城核心区、定鼎门等大遗址的保护展示，以及历史文化街区、工业文化遗产的保护展示，进一步提升城市文化品位。加强非物质文化遗产的发掘和保护，积极参与丝绸之路和大运河申报世界文化遗产工作。发展壮大文化产业。完善文化产业扶持政策，出台文化产业发展规划。重点发展现代传媒、演艺娱乐、文博会展等新兴产业，促进文化与其它产业的融合。大力发展文化产业集群，壮大唐三彩、澄泥砚、牡丹画等特色文化产业。积极发展文化创意产业，努力打造知名网络动漫品牌。开发各类衍生文化产品，形成新的文化产业链。加快龙门、汉魏故城、隋唐遗址、西霞院等文化旅游产业园区建设，做好龙门申报省级文化产业示范园区工作。大力培育重点文化企业，加快组建洛阳报业集团。大力发展文化事业。围绕河洛文化、丝路文化、牡丹文化、杜康文化，努力打造体现地域特色和时代精神的文化精品。加快匾额博物馆、丝绸之路博物馆、河南壁画馆等基础设施建设，建成并免费开放洛阳博物馆新馆。抓好新区图书馆、洛阳电视台演播中心等重大项目的前期工作。加强基层公共文化设施建设，实施农村数字电影放映工程和艺术团体百场公益性演出工程。广泛开展广场文化狂欢月、全民读书月等公益文化活动。加快推进文化体制改革。抓住我市被列为省文化体制改革试点市的机遇，分类推进文化单位改革。拓宽文化产业融资渠道，鼓励社会力量兴办文化经营实体。采取政府购买文化产品等措施，加快培育优势文化企业。

（九）做好财政金融工作，切实增强保障能力。大力组织财政收入。依法加强税收征管，规范非税收入管理，努力做到应收尽收。优化财政支出结构。重点加大对教育、卫生、社会保障、保障性安居工程等民生领域的支出，确保法定支出和省定、市定实事的需要。从严控制一般性支出，努力降低会议、接待、购车、出国等行政开支。深化财税体制改革。扩大国库集中支付、政府采购、投资评审等改革，实现政府公共预算、政府性基金预算和社会保障预算的有机衔接。加大金融支持力度。全面落实金融促进经济发展的政策措施，每季度组织召开不同层面的银企洽谈会，鼓励金融机构加大信贷投放力度，对有市场、有竞争力、发展前景好的企业给予支持，引导企业与金融机构建立稳定的合作关系。继续引进域外金融机构入驻洛阳。加快中信重工、北玻等企业上市步伐，鼓励上市公司再融资。支持有实力的企业发行债券，扩大信托融资规模。完善中小企业信用担保体系，引导社会资本投资设立担保机构。加快市商业银行改革和农村合作银行组建步伐，扩大村镇银行和小额贷款公司试点。

（十）加强和谐社会建设，让人民群众得到更多实惠。全力办好省定、市定实事。今年市财政将安排20亿元，切实解决关系人民群众切身利益的民生问题。各级各部门要进一步明确责任，强化措施，真正把实事办实、好事办好。认真做好就业再就业工作。全面落实社保补贴、税费减免等就业再就业优惠政策，全年发放小额担保贷款2亿元，新增城镇就业10万人。新开发公益性岗位1000个，确保零就业家庭至少有一人就业。鼓励劳动密集型产业特别是中小企业和服务业积极吸纳就业，引导大中型企业不裁员或少裁员。搞好就业培训、信息服务，为大中专毕业生、复转军人、待岗失业人员、失地农民创造良好的就业环境。落实农民工回乡创业各项优惠政策，大力支持农民工自主创业。完善城乡社会保障体系。加大社会保险扩面征缴和基金清欠力度，扩大企业养老保险、城镇职工基本医疗保险、城镇居民基本医疗保险和新型农村合作医疗的覆盖面。积极争取农村养老保险试点，加快实施失地农民社会保障制度。完善城乡社会救助体系和优抚保障制度，实现动态管理下的应保尽保。加大保障性住房建设力度，开工经济适用房32万平方米、廉租房14万平方米。国有大企业困难职工住房改造开工30万平方米、竣工15万平方米。改造农村困难群众危房7950间。加快发展社会事业。巩固提高义务教育，发展高中阶段教育，提高农村中小学生人均公用经费标准。加快学校标准化建设，改造农村初中和市直薄弱学校15所。开工建设2所新区初中。大力发展职业教育，提高劳动者素质。完善教育资助体系，认真解决农村特困户、城市低收入群众和农民工子女上学问题。落实教师绩效工资制度，切实提高教师待遇。加大公共卫生经费投入，加强基层卫生网络建设，改造标准化村卫生室1000个。推进优势医疗机构倍增计划，完善医疗服务体系和疾病控制体系。稳定低生育水平，综合治理出生人口性别比偏高问题。广泛开展全民健身活动，办好市第十一届运动会。全力维护社会安全稳定。加大对煤矿、非煤矿山、交通运输、尾矿库、人员密集场所等重点领域安全隐患的排查、治理力度，坚决遏制重特大安全事故发生。深入开展食品药品安全专项整治，确保人民群众食品药品安全。认真落实信访工作责任制，搞好矛盾纠纷排查化解，有效防范和处置群体性事件，着力维护群众合法权益。深入开展平安洛阳创建活动，加强治安防控体系建设，增强群众的安全感。加强精神文明建设。深入开展文明城市创建活动，着力打造诚信洛阳、礼仪洛阳、魅力洛阳。落实优抚安置政策，搞好双拥共建，支持国防和军队建设。依法保护妇女儿童、残疾人和未成年人合法权益。同时，继续做好对口支援江油灾后重建工作。

（十一）深入开展“两转两提”活动，加强政府自身建设。牢固树立执政为民的理念，进一步提高行政效能和服务水平，努力建设人民满意政府。加快转变政府职能。深化行政管理体制改革，加快推进政企分开、政事分开、政府与中介组织分开。争取上半年完成市级政府机构改革，年底前完成县级政府机构改革。加快事业单位分类改革。完善应急管理体系，提高应对危机和抗风险能力。提高依法行政水平。严格执行人大及其常委会的决议和决定，自觉接受市人大及其常委会的法律监督、工作监督和政协的民主监督，认真办理人大代表议案、建议和政协委员提案。完善沟通协商机制，广泛听取人大代表、政协委员和民主党派、工商联、无党派人士的意见，充分发挥各人民团体的桥梁纽带作用。完善立法机制，提高立法质量。加强行政执法培训，进一步提高执法水平。加强政府信用建设，提高政府公信力。不断提高行政效能。加快行政审批制度改革，取消和调整一批审批项目，加强对已取消审批项目的监管。优化项目审批程序，提高集中审批办结率。认真落实请办事项限时办结、首席服务官等优化环境的长效机制，做到多服务、少检查，多指导、少罚款。落实《政府信息公开条例》，提高政府工作的透明度。深入开展学习实践科学发展观主题教育活动，不断提高各级公务员推动科学发展的能力。进一步转变作风，深入基层，调查研究，做到问政于民、问需于民、问计于民。建立绩效考核评价机制，加大行政问责力度。着力打造廉洁政府。完善惩治和预防腐败体系，加大行政监察、审计力度，行政权力运行到哪里，监察就跟踪到哪里。严肃查处损害企业、投资者和群众利益的违法乱纪行为，重点解决环境保护、安全生产、土地征用与房屋拆迁等方面群众反映强烈的问题。

寄语洛城风日道，今年春色倍还人。各位代表！建设一个更温馨、更和谐、更美丽、更富强的新洛阳，是650万洛阳人民的共同愿望，也是各级政府义不容辞的责任。历史激励着我们，时代召唤着我们，人民期待着我们。让我们牢记使命，团结奋进，共同开创洛阳更加美好的未来！
